# In-Host Emergence of Linezolid Resistance in a Complex Pattern of Toxic Shock Syndrome Toxin-1-Positive Methicillin-Resistant *Staphylococcus aureus* Colonization in Siblings with Cystic Fibrosis

**DOI:** 10.3390/toxins13050317

**Published:** 2021-04-28

**Authors:** Agathe Boudet, Alexandre Jay, Catherine Dunyach-Remy, Raphaël Chiron, Jean-Philippe Lavigne, Hélène Marchandin

**Affiliations:** 1 VBIC, INSERM U1047, Université de Montpellier, Service de Microbiologie et Hygiène Hospitalière, CHU Nîmes, 30029 Nîmes, France; agathe.boudet@chu-nimes.fr (A.B.); catherine.remy@chu-nimes.fr (C.D.-R.); jean.philippe.LAVIGNE@chu-nimes.fr (J.-P.L.); 2 Service de Microbiologie et Hygiène Hospitalière, CHU Nîmes, 30029 Nîmes, France; alex01570@gmail.com; 3 HydroSciences Montpellier, Centre de Ressources et de Compétences de la Mucoviscidose, Université de Montpellier, CNRS, IRD, CHU de Montpellier, 34093 Montpellier, France; r-chiron@chu-montpellier.fr; 4 HydroSciences Montpellier, CNRS, IRD, Service de Microbiologie et Hygiène Hospitalière, CHU Nîmes, Université de Montpellier, 34093 Nîmes, France

**Keywords:** *Staphylococcus aureus*, cystic fibrosis, linezolid, resistance, emergence, siblings, diversity, adaptation, Geraldine clone, toxic shock syndrome toxin 1

## Abstract

Methicillin-resistant *Staphylococcus aureus* (MRSA) can cause chronic lung infections in patients with Cystic Fibrosis (CF). One option for managing them is the use of linezolid. We hereby report the in-host emergence of linezolid resistance (LR) in MRSA in CF siblings via a population analysis. A collection of 171 MRSA strains from 68 samples were characterized by determining their linezolid Minimal Inhibitory Concentrations (MICs), analyzing the locus of staphylococcal protein A (*spa*) and whole genome sequencing. Courses of linezolid were retraced. Strains belonged to three *spa* types (t002, t045, t127) and two sequence types (ST1, ST5). Emergence of LR occurred under treatment, one year apart in both siblings, in the CC5-MRSA-I Geraldine clone harboring the toxic shock syndrome toxin-1-encoding gene. Resistance was related to a G2576T substitution present in a variable number of 23S rRNA gene copies. Susceptible and resistant strains were co-isolated within samples. Single Nucleotide Polymorphism-based analysis revealed complex colonizations by highly diversified, clonally related populations. LR remains rare in MRSA and there are very few longitudinal analyses documenting its emergence. Analyzing a large MRSA collection revealed new aspects of LR emergence: it emerges in specific subclonal lineages resulting from adaptive diversification of MRSA in the CF lung and this heterogeneity of intra-sample resistance may contribute to compromising antibiotic management.

## 1. Introduction

Cystic fibrosis (CF) is a genetic disorder marked by a multi-organ disease due to a defect in the CFTR (cystic fibrosis transmembrane conductance regulator) protein, a transmembrane ion channel. Alteration in transmembrane ion exchanges result in abnormal mucus decreasing microbial clearance and creating local conditions favorable to bacterial persistence. In the respiratory tract, a cycle of colonization/infection and inflammation is established, leading to lung damage which has been identified as being responsible for morbidity and mortality in CF patients. 

The most frequently identified microbial species in CF patients is *Staphylococcus aureus*, with 70% of patients in the United States having at least one bacterial culture during the year 2019 and 62% in France during 2018 [1,2]. Regarding multidrug-resistant *S. aureus* strains, known as methicillin-resistant *S. aureus* (MRSA), a clear picture has emerged between the US and Europe, where 25% of the patients in the US had at least one MRSA isolate in 2019 compared to the lower prevalence observed in Europe (6% in France in 2018) [1,2]. This could be related to the spread of community-acquired MRSA clones in the general population from these two distinct continents.

MRSA can establish different types of lung colonization, i.e., transient, intermittent or chronic colonization. As detecting MRSA in the respiratory tract of CF patients is associated with lower lung function [3] and worse survival [4], and persistent infection is associated with a faster decline in the lung function [5], eradication is currently recommended. However, due to the antimicrobial multidrug-resistance patterns of MRSA, the options for antibiotic treatment are limited and there are no current recommendations or guidelines specific to the management of MRSA infections in CF. These usually combine antibiotics, topical decontamination and environmental cleansing. Antibiotic choices for MRSA infections include cotrimoxazole, rifampicin, fusidic acid (not licensed in the United States), minocycline and vancomycin (possibly inhaled), used either alone or in combination [6]. Linezolid, belonging to the oxazolidinone family, was discovered in the 1990s. It was first approved for use in 2000 and has been available in France since 2002. The drug inhibits the early stages of bacterial protein production after fixation on the 50S ribosomal subunit (domain V region of the 23S ribosomal RNA (rRNA) gene), and is also thought to inhibit the expression of virulence factors and decrease toxin production. Based on its action on MRSA, it was soon used for the successful treatment of pulmonary exacerbations due to MRSA in CF patients [6,7,8]. 

Rapidly, and as classically observed for many new antibiotics, studies reported the emergence of linezolid-resistant *S. aureus* (LRSA) in the general population in 2001 and in people with CF in 2004 [9]. Several mechanisms were associated with resistance to linezolid, all affecting the linezolid binding site. These included mutations in domain V of one or more of the copies of the 23S rRNA (*rrl*) gene, with G2576T being the most frequently observed mutation, mutations or deletions in the L3 and/or L4 ribosomal proteins, and acquisition of the plasmid-borne *cfr* gene, a multidrug-resistance gene encoding a 23S rRNA methylase. To date, resistance to linezolid is still considered exceptional, as fewer than 1% of MRSA strains worldwide are found to resist against linezolid [10]. Similar observations have also been made in the context of CF [11,12].

We hereby describe the in-host emergence of linezolid resistance in siblings with CF treated by linezolid through a populational analysis. Phenotypic and genotypic characterization of successively recovered MRSA strains revealed complex MRSA colonization and new aspects of the emergence of linezolid resistance in CF compared with the available literature.

## 2. Results

### 2.1. Patient Follow-Up and Microbiological Data

**Figure 1:**
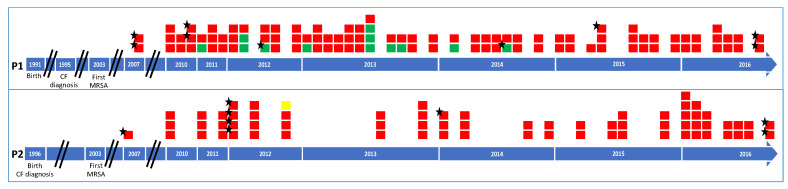
Timeline of methicillin-resistant *Staphylococcus aureus* (MRSA) strains included in the study for Patient 1 (P1) and Patient 2 (P2). Each square represents an MRSA strain included in the study (171 strains: 102 from Patient 1 and 69 from Patient 2). The piled-up squares represent strains with different colonial morphotypes within a sample (68 samples: 44 for Patient 1 and 24 for Patient 2). Strains are presented according to their genotype in the *spa* locus and MLST analyses (one color per profile as follows: red, *spa* type t002/ST5; green, t045/ST5; yellow, t127/ST1). Stars located on the top left corner of the boxes indicate strains with whole genome sequence analyzed in the study. Birth date, date of CF diagnosis and date of first MRSA isolation are indicated on the figure.

MRSA were isolated from all routinely sampled sputum specimens analyzed for both patients over the 2010-2016 period (45 and 46 samples for Patients P1 and P2, respectively). The isolation timeline of the 171 available MRSA strains (102 and 69 for patients P1 and P2, respectively) included in the study is presented for Patient 1 and Patient 2 in Figure 1, respectively, together with patient data (birth year, year of CF diagnosis, year of first MRSA colonization). Included MRSA had been isolated from 44 and 24 routinely sampled sputum specimens, respectively; two to four colonial morphotypes per sample were studied for Patient 1, and one to five colonial morphotypes per sample were studied for Patient 2.

### 2.2. Antimicrobial Susceptibility Profile and Linezolid Resistance

**Figure 2:**
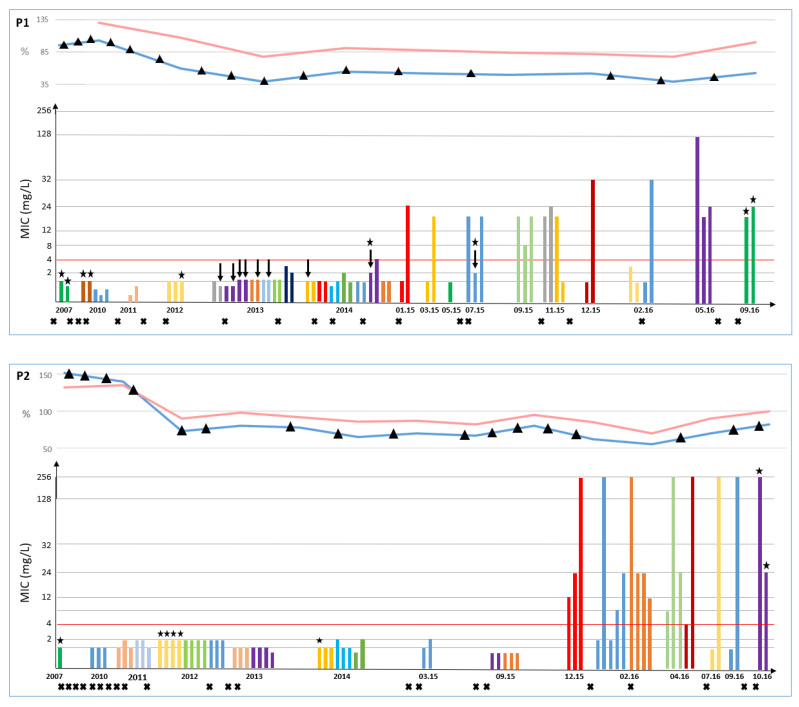
Susceptibility of linezolid, lung function parameters and episodes of pulmonary exacerbation over chronic colonization by methicillin-resistant *Staphylococcus aureus* (MRSA) in Patient 1 (P1) and Patient 2 (P2). Minimal Inhibitory Concentrations (MICs) of linezolid were determined by the Etest method and are reported for 132 MRSA strains isolated in Patient 1 (66 strains from 30 samples) and Patient 2 (66 strains from 23 samples) over a 9-year period of lung colonization. The year or detailed dates (MM/YY) of MRSA isolation are given in the figure. The red horizontal line indicates the susceptibility breakpoint according to antibiogram committee of the French Society for Microbiology (CA-SFM)/European Committee for Antibiotic Susceptibility Testing (EUCAST) standards for *Staphylococcus* (4 mg/L) [13]. Each color indicates strains that were isolated from a sample. Each cross at the bottom of the figure indicates a linezolid course. For P1, the black arrows indicate strains of *spa* type t045 (all other strains included in this figure are of *spa* type t002). Stars located above the bars indicate strains with whole genome sequence analyzed in the study. Lung function parameters shown are forced vital capacity (FVC (%), red curve) and forced expiratory volume in one second (FEV_1_ (%), blue curve). Each episode of pulmonary exacerbation is indicated by a triangle on the FEV_1_ curve.

**Figure 3:**
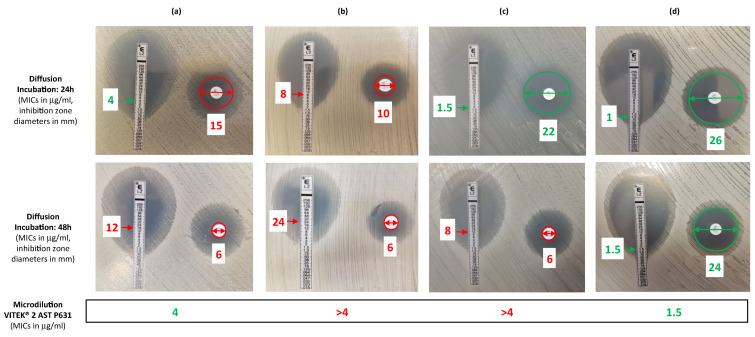
Comparative results for susceptibility to linezolid according to method (Etest of disk diffusion, microdilution using VITEK^®^ 2) and time of incubation for diffusion method (24 h or 48 h of incubation). (**a**) Results obtained for the first linezolid-resistant *S. aureus* (LRSA) isolated in December 2015 in Patient P2; (**b**) Results obtained for the first LRSA isolated in January 2015 in Patient P1; (**c**) Results obtained for the LRSA strain isolated in September 2015 in P1; (**d**) Results obtained for a linezolid-susceptible strain isolated in May 2016 in P1. Strains in (**a**,**c**) were selected to exemplify the discordance in linezolid susceptibility observed during the study depending on the method and incubation time used comparatively with strains in (**b**,**c**), classified as resistant and susceptible to linezolid whatever the method and time of incubation, respectively. Minimal inhibitory concentration (MIC) obtained using the VITEK^®^ 2 AST P631 card are indicated for each strain at the bottom of the figure. Inhibition zone diameters (in mm) and MIC values (in μg/mL) are indicated for each assay with a color code referring to the CA-SFM/EUCAST breakpoints (green = susceptible; red = resistant: MICs > 4 mg/L or inhibition zone diameters < 21 mm) [13].

All strains were MRSA displaying resistance to erythromycin, lincomycin, kanamycin, tobramycin, fusidic acid and rifampicin, and susceptibility to pristinamycin, gentamicin, minocycline, ofloxacin, fosfomycin, cotrimoxazole, ceftobiprole and ceftaroline, a profile compatible with the *tst*+ Geraldine clone to which the strains were affiliated. Linezolid resistance was the only modification detected in the antimicrobial susceptibility pattern over time and was first detected in January 2015 for Patient 1 (a 14-year-old) and in December 2015 for Patient 2 (a 10-year-old). Patients 1 and 2 had previously received thirteen and seventeen 15-day courses of linezolid, respectively. After first emergence, LRSA strains were identified in all subsequent samples included in the study. Testing up to five cultural morphotypes per sample revealed that linezolid-susceptible strains were co-isolated with LRSA strains in 10 out of the 18 samples including resistant strains (6/10 and 4/8 in Patients P1 and P2, respectively), as was observed the co-isolation of strains with distinct levels of linezolid resistance (Figure 2). For some strains, it should also be noted that discrepant results were observed between methods (Etest, disk diffusion and/or automated dilution technique) and that detection of linezolid-resistance may be detected after 48h of incubation only (Figure 3). The emergence of linezolid resistance did not modify the lung function or the rate of pulmonary exacerbation (Figure 2).

### 2.3. Strain Genotyping

The *spa* screening approach showed that MRSA strains presented three distinct profiles. *spa* typing showed that these three profiles corresponded to three *spa* types (t002, t045, t127), and MLST/wgMLST confirmed that these three *spa* types belonged to 2 STs (ST5 for t002 and t045, ST1 for t127). According to the Ridom SpaServer, the three *spa* types identified in our study had previously been reported in different countries and their frequencies were 6.57% for t002 (the third most common *spa* type in the database), 2.63% for t127 and 0.69% for t045 (http://spa.ridom.de/frequencies.shtml, accessed on 20 February 2021). MRSA of t002 were the most identified in both patients, representing 85% of strains in the study (86% in Patient 1 and 98% in Patient 2). Patient 1 presented a co-colonization by MRSA of two related *spa* types, t002 and t045 with t045 (repeat succession 26-17-20-17-12-17-16), presenting a deletion of three repeats compared with t002 (repeat succession 26-23-17-34-17-20-17-12-17-16). 

One MRSA of t127/ST1 was found in Patient 2 but did not manage to colonize as it was only identified once. Regarding the linezolid susceptibility of the three MRSA types identified, resistance was observed in t002 strains only (Figure 2).

### 2.4. Identifying of the Genetic Determinant of Linezolid Resistance

Four LRSA strains (two strains co-isolated from the last sample included in the study for each of the two patients and showing linezolid MICs of 16, 24, 32 and >256 mg/L) were submitted to whole genome sequencing to determine the genetic determinant(s) of linezolid resistance. Whole genome sequence-based analysis identified a unique mutation (G2576T substitution) in the sequences of the four LRSA strains analyzed, whereas this substitution was not found in any of the 13 WGSs analyzed for susceptible strains, either of t002 or t045 (data not shown). 

I-*Ceu*I macrorestriction-based analysis of the DNA of seven strains (the three susceptible strains isolated in 2007 and the four LRSA strains isolated in 2016) revealed that all possessed five copies of the 23S rRNA gene (data not shown). Depending on the strain, three or four of the five copies of the 23 rRNA gene harbored the G2576T substitution (Supplementary Table S1). Strains with MICs of 16, 24 or 32 mg/L of linezolid harbored three mutated copies and the strain with MIC >256 mg/L harbored four mutated copies. *rplD* (encoding the L4 ribosomal protein) and *rplC* (encoding the L3 ribosomal protein) genes were wild type and the *cfr* gene was not detected.

### 2.5. Relationship between Isolates

The SNP-based analysis revealed that whole genome sequences (WGSs) of the 9 strains analyzed for Patient 1 presented 12 to 71 SNPs, whereas those of the 8 strains analyzed for Patient 2 presented 41 to 135 SNPs. On an inter-patient level, 18 to 121 SNPs were found between WGSs (Table 1). According to the study by Ankrum et al., all strains were considered as clonally related, including strains of t002 and t045 in Patient 2, and the SNPs observed were considered as corresponding to intra-host diversification.

One strain isolated in Patient 1 in 2010 had over 1000 SNPs with other strains isolated and was thus considered as a superinfecting strain, unrelated to the persistent clone in this patient. The latter strain was not detected using *spa* screening showing that our approach may have underestimated the diversity of strains. 

In total, we considered that all other isolates shared a common ancestor. In both patients, an irregular but overall increase in the number of SNPs was observed over time, as was the observed genetic diversity among strains co-isolated within a sample (Table 1).

## 3. Discussion

Linezolid resistance remains a rare finding in CF. We identified 13 previously published studies in this setting, reporting a total of 34 patients with LRSA (of which two described linezolid-dependent strains) [9,11,12,15,16,17,18,19,20,21,22,23,24]. The only multicentric study currently available includes 277 MRSA strains from individual patients attending seven pediatric CF centers in the USA and showed that 0.4% were LRSA [12]. In 2011, also in the USA, Endimiani et al. reported that 2% of patients (8/390) attending their center over a 7-year period had at least one respiratory specimen culture with an LRSA isolate [11]. LRSA was more rarely reported in European countries (10 CF patients with LRSA), probably due to the distinct epidemiology and the less significant rate of CF patients colonized by MRSA in Europe (e.g., 6% in France versus 25% in the USA). In Europe, LRSA was mostly observed in Spain and Italy, whereas our study is the third one reporting LRSA in CF patients in France [17,23].

In most studies, the level of resistance to linezolid is associated with the mechanism of resistance. A higher level of resistance has been observed along with the number of mutated *rrl* gene copies [11]. Strains may also cumulate the different mechanisms associated with resistance to linezolid [19]. Here, we observed a heterogeneous level of resistance to linezolid depending on the strain and the patient: strains from one patient exhibited high MIC values >256 mg/L although not all 23S rRNA gene copies were mutated and this was in the absence of other identified resistance mechanisms, which is an uncommon observation. 

Similarly, distinct dynamics of resistance were observed between siblings with strains displaying high MICs >256 mg/L observed as soon as resistance emerged, whereas these were never described in Patient 2. Linezolid exposure has been shown to be significantly associated with the emergence of linezolid resistance in most—but not all—patients [11,21,25]. It is worth noting that when Endimiani et al. restricted their study to patients who had received linezolid, the rate of patients with LRSA reached 10.4% [11]. In our report, both siblings had received several courses of linezolid resulting in roughly similar pressure before the emergence of resistance (195 and 255 cumulated treatment days, respectively). In the literature, we found that the time of emergence greatly varied according to studies, showing that multifactorial determinants for emergence are probably involved in a specific host/*S. aureus* partnership. Selection of LRSA was indeed observed as early as after 13 days of treatment [17,18] to a mean time of 128 days of treatment (range: 24–453 days) in the largest study by Yu et al. reporting on 11 patients with LRSA [21]. One of the factors favoring the selection of LRSA clearly identified in previous studies is the failure to reach adequate pharmacokinetic/pharmacodynamic conditions, a particularly difficult point with CF and pediatric patients [9,11,15,26]. In our study, there was no evidence of failure to comply with treatment by the two patients, but no linezolid dosing was performed during the study period.

In our retrospective study, we noted that linezolid resistance had been overlooked for these patients, both siblings having received linezolid after the emergence of LRSA due to non-detection of resistance in routine practice. A combination of several concomitant factors contributing to this non-detection deserve special attention: (i) a non-systematic testing of linezolid in the antimicrobial susceptibility testing panel at the time of the study (the transfer of linezolid from the complementary to the standard panel of antibiotics to be systematically tested in the antibiogram committee of the French Society for Microbiology (CA-SFM) recommendations for *S. aureus* only became effective in France in September 2018), (ii) a complete antibiogram was only performed once a year in chronic MRSA infections, (iii) the difficulty of detecting resistance as explained herein, and (iv) the intra-sample heterogeneity definitely proven in this work and discussed later. Although the recommendations of the CA-SFM are to carefully examine the inhibition zone and include a warning to say that detection of inducible resistance may require prolonged incubation and a reading after 48h of incubation [13], *S. aureus* disk diffusion antimicrobial susceptibility assays are usually read after 24 h of incubation and this may account for the non-detection of resistance to linezolid as exemplified herein. Although the study was not pursued after the end of 2016 for either patient, as there were no further courses of linezolid, both patients are still colonized by LRSA, a congruent observation with the persistence of LRSA described in previous studies even in the absence of linezolid exposure [15,23].

Resistance to linezolid has more often been reported among clones that are endemic to the corresponding reporting country, such as the Spanish clone (ST125-*spa* type t067-SCC*mec* Ivc) [19,20], the Brazilian MRSA clone [9], the highly prevalent EMRSA-16 (ST36-MRSA-II) clone in the UK [15], or the USA100 clone (ST5/SCC*mec* II), also known as the New York/Japan clone [11,18]. Here, we report the first cases of linezolid resistance in strains belonging to the MRSA Geraldine clone which contains the *tst* gene encoding for toxic shock syndrome toxin-1 (TSST-1). This *tst*+ MRSA clone is an epidemic clone of ST5 genetic background which harbors a peculiar, truncated SCC*mec* Type I cassette and occurs mainly in young people in whom it is responsible for a wide diversity of clinical syndromes including toxin-mediated and suppurative diseases [27]. It is responsible for both hospital- and community-acquired infections and represents a minor clone among invasive MRSA strains in France [28]. MRSA isolates that carry the TSST-1-encoding gene have been extremely rarely reported in CF [29] and their role in disease evolution and patients’ clinical status have yet to be investigated.

Although our study has certain limitations, including those inherent to retrospective studies and the fact that not all the strains were analyzed by WGS, its main originality is that it includes the study of several strains per sample over a long period of time. Indeed, longitudinal studies of this kind are rare in the literature [15,17,19,23] and none of them included as many strains per patient and several strains per sample. Despite this, we have probably not characterized the entire diversity of MRSA in the siblings included as shown by the unrelated strain in WGS showing *spa* type t002 identical to other colonizing strains. Nevertheless, the population analysis we conducted has revealed a complex, dynamic colonization by a phenotypically (susceptibility/resistance to linezolid) and/or genotypically (*spa* types, SNPs) highly diversified MRSA population. Such populations encompassing a wide variety of subclonal variants, including variants displaying a striking diversity of antimicrobial resistance patterns, have been largely reported for Gram-negative pathogens during chronic colonization in the lungs of CF patients [30,31,32,33,34]. They reflect an adaptive strategy developed by CF pathogens for long-term survival in the CF lung, allowing them to quickly adapt and face fluctuating selective pressures, the so-called “insurance hypothesis” [35]. For *S. aureus*, diversified populations have been more rarely documented in CF [14,36,37,38,39] and population heterogeneity has only once been related to antimicrobial susceptibility patterns [36]. Here, we documented intrasample heterogeneity of susceptibility to linezolid, which may explain some of the previous observations (alternate detection of susceptible and resistant strains, non-chronological accumulation of SNPs) made by other authors [14,17,23]. This was only related to linezolid emergence observed in the subpopulation of t002 in Patient 1, whereas the variant of t045 remained susceptible despite similar exposure to linezolid. It is currently unknown whether some MRSA genetic backgrounds or lineages may lead to linezolid resistance emergence faster and this point warrants further investigations.

In siblings with LRSA strains, differentiating cross-transmission of the resistant strain (which had emerged in one of the patients and was further transmitted between siblings) from the emergence of resistance in the MRSA colonizing each patient is highly challenging. We reexamined the results of three previous studies that included siblings [11,19,21]. Caballero et al. noted that their results may indicate a cross-transmission event between siblings, as they found that the isolates had identical genotypes and linezolid resistance levels and determinants [19]. Similarly, Yu et al. identified LRSA at the same date in siblings with significant different exposure to linezolid that more likely reflects cross-transmission of LRSA [21]. However, Endimiani et al. reported a more complex picture of LRSA colonization in siblings with long-term exposure to linezolid and LRSA detection 10 months apart. Indeed, strains displayed the same resistance mechanism, i.e., G2576T mutation; however, the mutation was observed in a distinct number of *rrl* gene copies. Interestingly, the G2576T mutation combination was unique to each patient (i.e., the mutated copy observed in the first patient detected with LRSA was not part of the three mutated copies observed in the second patient). Although strains shared identical PFGE profiles and related *spa* types t002 and t2051, the overall results suggested a distinct within-host evolution of related strains through homologous recombination of wild-type and mutated *rrn* genes producing the different numbers and locations of the G2576T mutation observed while not excluding a previous event of cross-transmission [11]. In our study, that is, the only one including WGS analysis to compare LRSA strains from siblings, strains isolated in 2016 were considered as “identical” or “very closely related” between siblings. However, a similarly close relationship had already been observed between strains isolated in siblings in 2007 making it difficult to distinguish linezolid resistance emergence in each of the two patients from their colonizing strains from a cross-contamination event that apparently occurred after resistance emergence in the first sibling during the year 2015. However, we noted distinct diversification rates in each patient, with all strains in Patient 1 showing ≤ 71 SNPs, whereas strains in Patient 2 diverged by up to 137 SNPs with an irregular but overall increase in SNPs over time in both patients, and also on an interpatient level (18-22 SNPs in 2007, 39-74 SNPs in 2012, 101 in 2014, 43-82 in 2016). Although we were unable to trace and date all the potential cross-transmission events between siblings, LRSA strains seemed to have emerged from strains that had differentially evolved in both hosts.

## 4. Conclusions

Still rarely described in CF, linezolid resistance must be carefully monitored in CF patients chronically colonized by MRSA. This included a regular antibiotic susceptibility testing, particularly for drugs used in the management of these patients. The bacterial populations colonizing these patients are highly complex and dynamic, particularly in patients receiving linezolid whether for anti-MRSA purposes or as part of the antimicrobial regimen against nontuberculous mycobacteria.

## 5. Materials and Methods

### 5.1. Patients, Bacterial Strains and Data Collection

Two CF siblings (Patients P1 and P2) attending the CF center at Montpellier University Hospital, France, were included in this study. Ethical approval was obtained through the Institutional Review Board at Nîmes University hospital (Interface Recherche Bioéthique IRB n 21.03.01, 04 March 2021). Both patients are chronically colonized by MRSA and *Pseudomonas aeruginosa*. These are two sisters whose genotype is F508del/R553X, born in 1991 (P1) and 1996 (P2), respectively. The oldest child had a childhood and adolescence without an antibiotic infusion, without bronchial obstruction and simply the use of oral antibiotics against *S.aureus*. There was a sharp decline of FEV_1_ from the age of 18, concomitant with *P. aeruginosa* colonization) (Figure 2). She is glucose intolerant and has exocrine pancreatic insufficiency. Her younger sister had a much stormier childhood with quarterly infusions since the age of 10 for bronchial exacerbations and severe obstructive disorder (first *P. aeruginosa* colonization at the age of 12). She is diabetic without the need for insulin and has exocrine pancreatic insufficiency.

MRSA bacterial strains isolated from routinely sampled sputum specimens and analyzed as part of the standard follow-up of both patients were included. During this standard follow-up, antimicrobial susceptibility testing was performed once a year according to the National Diagnosis and Care Plan for Cystic Fibrosis for long-term chronically colonized patients by multidrug resistant bacteria [40]. Strains had been collected over a 7-year period (2010–2016) and stored frozen at −80 °C. Additionally, one strain from early infection episodes in each patient (2007) was included for the purpose of comparing them with strains isolated during the study period (these strains were the first ones available for P1 and P2). Both strains were previously analyzed by the microarray ADN Alere^®^ *Staphylococcus aureus* genotyping kit 2.0 (Alere Technologies, Jena, Germany) [41] showing them to belong to the CC5-MRSA-I Geraldine clone, described as *agr*-2, ST5, *spa* type t002 or related, and SCC*mec* type I, and harboring the *tst* gene [28]. Data regarding age at first MRSA isolation, co-colonizing pathogens and linezolid courses received by patients P1 and P2 were collected retrospectively.

### 5.2. Culture Conditions and Antimicrobial Susceptibility Testing

All strains were cultured on Trypticase Soy Agar with 5% Sheep Blood (bioMérieux, Marcy l’Étoile, France) and one colony was subcultured onto the same agar medium for all further analyses. 

Antimicrobial susceptibility testing was performed using disk diffusion and was interpreted according to the recommendations of the CA-SFM/EUCAST for *Staphylococcus* [13]. 

For linezolid, Minimal Inhibitory Concentration (MIC) was also determined by the Etest method (bioMérieux). According to the CA-SFM/EUCAST recommendations, resistance to linezolid is defined as MICs > 4 mg/L or inhibition zone diameters < 21 mm around a 10-μg disk, and inducible resistance may require a prolonged incubation and reading after 48 h of incubation [13]. Results of linezolid susceptibility assays (disk and Etest) were read after 24 and 48 h of incubation. For selected strains, susceptibility to linezolid was also investigated using the VITEK^®^ 2 AST P631 card for comparison with agar diffusion methods.

### 5.3. DNA Extraction, Analysis of the Staphylococcal Protein A (spa) Locus and Multi Locus Sequence Typing

DNA extraction was performed as previously described [42]. All strains were submitted to the amplification of the *Staphylococcus* protein A (*spa*) repeat region according to the *spa* typing website (http://www.spaserver.ridom.de/, accessed on 20 February 2021) developed by Ridom GmbH and curated by SeqNet.org (http://www.SeqNet.org/, accessed on 20 February 2021) [43]. Amplification products were subjected to electrophoresis for 2 h at 100 V in a 2% agarose gel in 0.5× Tris-Borate-EDTA gel. Amplification product size defines a *spa* profile that allowed a first stage of longitudinal screening of the entire strain collection. Selected strains (strains of each *spa* profile and multiple strains for each *spa* profile) were analyzed by *spa* typing as previously described to validate the *spa* screening approach [43]. Ridom StaphType™ software (Ridom GmbH, Würzburg, Germany) was used for *spa* sequence analysis. Sequence Type (ST) was determined for selected strains after Multi Locus Sequence Typing (MLST), either according to Enright et al. and Crisóstomo et al. [44,45] or after whole genome sequencing (wgMLST).

### 5.4. Determining the Rrn Copy Number

Macrorestriction of genomic DNA embedded in agarose plugs followed by pulsed-field gel electrophoresis was performed as previously described using I-*Ceu*I, an intron-encoded endonuclease that cleaves a specific 19-bp sequence in the 23S rRNA gene [46].

### 5.5. Whole Genome Sequencing

Seventeen whole genome sequences (WGSs) were selected among the WGSs available for MRSA strains of both patients as follows: nine WGSs for P1 (strains isolated from six samples between 2007 and 2016, including two LRSA isolated in 2016) and eight WGSs for P2 (strains isolated from four samples from 2007 to 2016, including two LRSA isolated in 2016). WGSs were obtained by an Illumina MiSeq platform (Illumina Inc., San Diego, CA, USA). Read quality was checked by FastQC [47], de novo assembled with SPAdes 3.12.0 [48] and annotated with Prokka 1.14.5 [49]. Pan-genome analysis was carried out using Roary 3.13.0 [50]. This whole genome shotgun project has been deposited at DDBJ/ENA/GenBank under the accession numbers JAGPWI000000000 to JAGPWK000000000 and JAGPWM000000000 to JAGPWZ000000000. The version described in this paper is version JAGPWI010000000 to JAGPWK010000000 and JAGPWM010000000 to JAGPWZ010000000. Sequence Read Archive accession number is PRJNA721116. Characteristics of the draft genome sequences are given in Supplementary Table S1.

LRE-finder was used to detect linezolid resistance genetic support and estimate the proportions of mutant vs. wild-type alleles in the 23S rRNA gene [51]. The latter data were used together with the number of 23S rRNA gene copies found in the strains under investigation to estimate the numbers of wild-type vs. mutated copies [52]. Core genome SNPs were called using Snippy [53] and SNPs numbers were interpreted according to the criteria of Ankrum et al., that define strains with ≤71 SNPs as the “same” strains, strains with 72 to 123 SNPs as “very closely related” strains, strains with 124 to 156 SNPs as “closely related” strains, and strains having ≥157 SNPs as “distantly related” strains [14].

## Figures and Tables

**Table 1:** Relationships between 17 methicillin-resistant *Staphylococcus aureus* strains isolated from 10 samples in Patient 1 and Patient 2 between 2007 and 2016 based on the number of single nucleotide polymorphisms observed in pairwise comparison of whole genome sequences.

		Patient 1									Patient 2							
		Jul. 2007		Oct. 2010		Jul. 2012	Oct. 2014	Jul. 2015	Sep. 2016		May 2007	Feb. 2012				Mar. 2014	Oct. 2016	
		t002	t002	t002	t002	t045	t045	t002	t002	t002	t002	t002				t002	t002	
		1	2	3	4	5	6	7	8	9	10	11	12	13	14	15	16	17
**Patient 1**	1		23	26	> 1.000	29	33	54	41	37	22	55	51	37	32	74	47	66
	2			43		45	49	69	57	54	18	42	38	54	49	62	67	80
	3					29	34	51	44	41	43	76	72	41	36	95	52	67
	4					>1000					>1000							
	5						11	51	49	52	46	78	74	42	39	97	57	73
	6							60	71	57	48	82	78	48	43	101	64	77
	7								71	68	71	103	100	68	63	121	79	97
	8									13	56	90	86	56	31	109	46	82
	9										53	87	83	53	28	106	43	79
**Patient 2**	10											53	47	54	49	72	64	82
	11												70	86	81	90	100	115
	12													81	80	87	95	111
	13														41	96	56	61
	14															101	27	76
	15																122	135
	16																	92
	17																	

Red font indicates linezolid-resistant strains. Cells were colored with a gray gradient according to the Ankrum criteria as follows: dark gray, ≤71 SNPs defining “same” strains; medium gray, 72 to 123 SNPs defining “very closely related” strains; light gray, 124 to 156 SNPs defining “closely related” strains [14].

## Supplementary Materials

The following are available online at https://www.mdpi.com/article/10.3390/toxins13050317/s1, Table S1: Characteristics of the 17 *Staphylococcus aureus* sp. draft genome sequences.
